# Pilot Trial Characteristics, Postpilot Design Modifications, and Feasibility of Full-Scale Trials

**DOI:** 10.1001/jamanetworkopen.2023.33642

**Published:** 2023-09-14

**Authors:** Xiangji Ying, Stephan Ehrhardt

**Affiliations:** 1 Department of Epidemiology, Johns Hopkins Bloomberg School of Public Health, Baltimore, Maryland

## Abstract

**Importance:**

Pilot trials often lead to study design changes in subsequent full-scale trials. Yet, it remains unclear whether these modifications improve the feasibility of the larger trial.

**Objective:**

To compare feasibility estimates between pilot and full-scale trials and identify pilot trial characteristics and modifications associated with equivalent or improved feasibility in the full-scale trial.

**Design, Setting, and Participants:**

This cohort study used pilot trials published between January 2005 and December 2018 and their corresponding full-scale trials. PubMed was searched for trials on February 19, 2022.

**Exposures:**

Pilot trial characteristics and postpilot trial design modifications.

**Main Outcomes and Measures:**

The outcome of interest was difference in 3 feasibility parameters: successful screening probability, enrollment rate, and retention probability. These metrics were defined as equivalent or improved if the full-scale trial’s estimate was within or exceeding 10% of the pilot trial’s estimate.

**Results:**

A total of 249 pairs of pilot and full-scale trials were analyzed, with 43%, 77%, and 82% of full-scale trials having equivalent or improved successful screening probabilities, enrollment rates, and retention probabilities, respectively. When pilot trials used feasibility progression criteria (relative risk [RR], 1.94; 95% CI, 1.02-5.97) and maintained masking for participants (RR, 1.82; 95% CI, 1.04-4.33) or health care practitioners (RR, 1.81; 95% CI, 1.03-3.97) consistent with the full-scale trial, the likelihood of achieving equivalent or improved screening success in full-scale trials increased. Increasing study sites after the pilot was associated with higher likelihood of equivalent or improved enrollment rates (RR, 1.03; 95% CI, 1.01-1.08). Adding extra content to the intervention (RR, 0.82; 95% CI, 0.66-0.98), changing to active control (RR, 0.74; 95% CI, 0.48-0.99), administrating the control treatment more frequently (RR, 0.60; 95% CI, 0.29-0.93), different follow-up lengths (RR, 0.85; 95% CI, 0.73-0.97), and more follow-up visits (RR, 0.86; 95% CI, 0.75-0.98) were associated with lower likelihood of equivalent or improved retention probability in the full-scale trial.

**Conclusions and relevance:**

In this cohort study of pilot and full-scale trial pairs, pilot trial characteristics and postpilot modifications had varying associations with full-scale trial’s feasibility. If full-scale trials planned for masking, it was desirable to use it in the pilot. Modifications increasing participant burden might decrease full-scale trial feasibility. Trialists and funders should consider both pilot trial data and proposed design changes when assessing full-scale trials.

## Key Points

**Question:**

What pilot trial characteristics and postpilot trial modifications are associated with improved feasibility in full-scale trials?

**Findings:**

This cohort study analyzed 249 pilot and full-scale trial pairs. Using feasibility progression criteria in pilot trials and maintaining the same masking status as the full-scale trial was associated with increased rates of successful screening, whereas adding extra content to the intervention, changing to active or more frequent control, and altering follow-up lengths and visits was associated with decreased rates of retaining participants in full-scale trials.

**Meaning:**

In this study, various pilot trial characteristics and postpilot modifications were associated with full-scale trial feasibility, warranting careful consideration during feasibility assessments.

## Teaser

This cohort study compares feasibility estimates between pilot and full-scale trials and identifies pilot trial characteristics and modifications associated with equivalent or improved feasibility in the full-scale trial.

## Introduction

In the past 2 decades, there has been growing attention on pilot studies. A basic PubMed search using the term *pilot study* yielded 668 articles in 2000 and 5484 articles in 2020. Traditionally, pilot studies served the purpose of evaluating feasibility and providing preliminary evidence on efficacy.^1^ However, the appropriateness of pilot studies in evaluating efficacy has been questioned due to their small sample sizes.^2,3,4,5,6^ It has been recommended that pilot studies should focus primarily on feasibility estimation, such as calculating probabilities of recruitment, randomization, intervention adherence, and attrition.^7^

Pilot trials are a specific type of pilot study that utilizes a randomized design.^8,9^ Although the emphasis is on using pilot studies or pilot trials for feasibility, few studies have examined the accuracy of their estimates in predicting parameters for full-scale trials. A recent empirical analysis of 16 pairs found that, on average, pilot trials provided variable but unbiased estimates for randomization and attrition probabilities.^10^ The authors speculated that the differences could be due to remedial action taken in the full trial to address issues identified in the pilot.^10^

It is not uncommon for trials to modify their designs after the pilot trial, as identifying areas requiring modification is one of the key objectives of conducting a pilot trial. A recent analysis found that 75% of full-scale intervention trials on obesity differed from the pilot trial in at least one domain, such as intervention intensity and implementation support.^11^ However, it is often unclear how those modifications will impact the feasibility of conducting the full-scale trial, especially when multiple aspects of the trial are being modified, which adds an extra layer of complexity. Ideally, a new pilot trial incorporating those changes would provide the most current feasibility data, but this comes with additional resource demands and potential delays in generating definitive evidence.^12^ Moreover, repeating this approach may not be practical should further modifications be required after the new pilot. This study therefore aims to compare feasibility estimates between pilot and full-scale trials and explore whether certain pilot trial characteristics and modifications are associated with equivalent or improved feasibility in full-scale trials.

## Methods

We followed the Strengthening the Reporting of Observational Studies in Epidemiology (STROBE) reporting guideline for cohort studies.^13^ Since the analysis was conducted at the study level without involving human participants, it did not require ethics approval or informed consent per the Common Rule.

### Literature Search and Study Selection

A systematic search in PubMed was conducted on February 19, 2022, to identify pilot trials published between January 2005 and December 2018. The search was restricted to English and included 3 concepts: pilot or feasibility study, randomized clinical trial, and feasibility parameters (eTable 1 in Supplement 1). A pilot study was defined as a small-scale investigation aimed at testing feasibility of methods for large-scale application or exploring potential effects and associations to be examined in a future larger study.^1^ Stand-alone pilot studies that utilized a randomized design were considered for inclusion. We used these inclusive early definitions to cover the timeline and to account for the varied use of the term pilot trial in literature.

To identify the subsequent full-scale trial that was conducted by the same research team and had an overlap in population characteristics with the pilot, we screened articles that cited the pilot trial. A full-scale trial was included if it had at least one group that was the same or similar to the pilot. We excluded the full-scale trial if it was informed by multiple pilot trials simultaneously.

### Data Extraction

We gathered information on trial characteristics, feasibility estimates, and efficacy estimates for each pilot and full-scale trial pair in Covidence using a form that had been pilot tested. Our selection of these characteristics was guided by research on factors influencing trial generalizability^14,15,16^ or participant recruitment and retention.^17,18,19,20,21,22,23^ To ensure data accuracy and minimize missingness, we extracted and cross-checked information from trial reports, protocols, and registries, prioritizing trial reports in case of discrepancies. Protocols were crucial for supplementary details when the trial report did not adequately describe elements, such as intervention procedures and outcome measurements. We compared the trial characteristics of the full-scale trials with their pilot trials to identify any modifications made to trial design, participant eligibility, intervention, control, and outcome measurement.

### Feasibility Parameters

The study examined 3 feasibility parameters. They were probability of successful screening, enrollment rate, and retention probability.

Successful screening means that a study participant is both eligible and willing to be randomized. We calculated the probability of successful screening by dividing the number of randomized participants by the total number of participants screened.

The enrollment rate was calculated by dividing the number of participants randomized by the duration of recruitment in weeks. A site-average rate was also computed by dividing this overall rate by the number of sites. Unless specifically mentioned otherwise, any reference to enrollment rate in this article pertains to the overall, not per-site, estimate.

For the probability of retention, we divided the number of participants who completed the study by the number of participants who were initially randomized. Noncompletion can be caused by competing events, withdrawal, loss to follow-up, and protocol deviations. To maintain consistency, we used the same definition of dropout within each pair of pilot and full-scale trials, as different studies had varying definitions. Whenever possible, we calculated the retention probabilities at the same time point in both the pilot and full-scale trials.

### Statistical Analysis

We described the feasibility estimates from pilot and full-scale trials using either the mean and SD or median and IQR if the estimate was heavily skewed. To evaluate the agreement between the pilot and full-scale trials, we calculated the percentage difference by dividing the difference between the 2 studies (ie, pilot − full-scale) by their mean.^24^

All pilot trials in our sample progressed to full-scale trials, indicating that the trialists deemed the full-scale trial feasible, either initially or after making protocol modifications. We considered the full-scale trial’s feasibility estimate to be equivalent if it fell within 10% of the pilot trial’s estimate, in either direction, or improved if it was more than 10% greater than the pilot trial estimate. We chose the 10% threshold because it accounted for possible random fluctuations and was commonly used in sample size calculations to adjust for dropouts. We used logistic regression to identify characteristics and modifications of pilot trials associated with equivalent or improved feasibility in the full-scale trial. The resulting odds ratios were converted to relative risk (RRs), and corresponding percentile-based confidence intervals were calculated using 10 000 bootstrap replications.^25^

All analyses were performed using Stata (Version 16; StataCorp, TX) and RStudio (Version 2022.12.0 + 353). A 2-sided *P* < .05 was considered statistically significant.

## Results

### Study Characteristics

A total of 249 pairs of pilot and subsequent full-scale trials were identified (eFigure 1 in Supplement 1). These pairs investigated a range of diseases (eTable 2 in Supplement 1), with most (172 [69%]) focusing on behavioral interventions (Table 1). Most pilot trials (186 [75%]) were conducted in a single center, while more than half (134 [54%]) of full-scale trials were multicenter. The proportion of trials with 2 groups was the same for both pilot and full-scale trials (210 [84%]). A mean (SD) of 121 (300; median [IQR], 53 [31-100]) individuals were randomized in pilot trials, while full-scale trials randomized a mean (SD) 1164 (4111; median [IQR], 264 [143-600]) individuals. The mean (SD) and median (IQR) follow-up duration in full-scale trials were approximately twice as long as in pilot trials (mean [SD], 321 [428] days vs 166 [237] days; median [IQR], 182 [91-365] days vs 91 [42-182] days).

**Table 1.** Key Characteristics Shared by Pilot Trials and Subsequent Full-Scale Trials

Characteristic	Trials, No. (%)	
	Pilot trial (n = 249)	Full-scale trial (n = 249)
Disease^a^		
Addiction	24 (10)	24 (10)
Mental health	34 (14)	34 (14)
Obesity and physical activity	27 (11)	27 (11)
Oncology	21 (8)	21 (8)
Other	143 (57)	143 (57)
Intervention		
Behavioral	172 (69)	172 (69)
Pharmaceutical & other	77 (31)	77 (31)
Publication year		
2004-2009	74 (30)	6 (2)
2010-2014	103 (41)	51 (20)
2015-2019	72 (29)	123 (49)
2020-2022	0 (0)	69 (28)
Funding source		
Nonindustry	220 (88)	230 (92)
Industry	6 (2)	12 (5)
None or not reported	23 (9)	7 (3)
Cluster randomization		
No	233 (94)	211 (85)
Yes	16 (6)	38 (15)
Sites		
Single center	186 (75)	115 (46)
Multicenter	63 (25)	134 (54)
Groups		
2	210 (84)	210 (84)
>2	39 (16)	39 (16)
Sample size		
Mean (SD)	121 (300)	1164 (4111)
Median (IQR)	53 (31-100)	264 (143-600)
Masking used		
No	139 (56)	90 (36)
Yes	110 (44)	159 (64)
Primary length of follow-up, d		
Mean (SD)	166 (237)	321 (428)
Median (IQR)	91 (42-182)	182 (91-365)
Intervention efficacy		
Not statistically significant	109 (44)	119 (48)
Statistically significant	92 (37)	129 (52)
Not evaluated	48 (19)	1 (<1)

^a^ The diseases listed represent the top 4 most frequently occurring within the data set. All other disease types are grouped under the category labeled as other. A complete list of diseases is available in eTable 2 in Supplement 1.

Data on successful screening probability, enrollment rate, and retention probability were available in 183, 177, and 238 pairs of pilot and full-scale trials, respectively. Comparisons of characteristics between pairs with and without missing data for these parameters are provided in the eAppendix and eTables 3 and 4 in Supplement 1.

### Successful Screening

The mean (SD) successful screening proportion among 183 trial pairs was 47% (27) for pilot trials and 41% (27) for full-scale trials. The mean (SD) percentage difference between pilot and full-scale trials was 15% (60; median [IQR], 14% [−21% to 47%]). As shown in the Figure, the percentage differences are symmetrically distributed around the mean, with a tendency for both the magnitude and variability to decrease as the sample size of the pilot trial increased.

**Figure.**
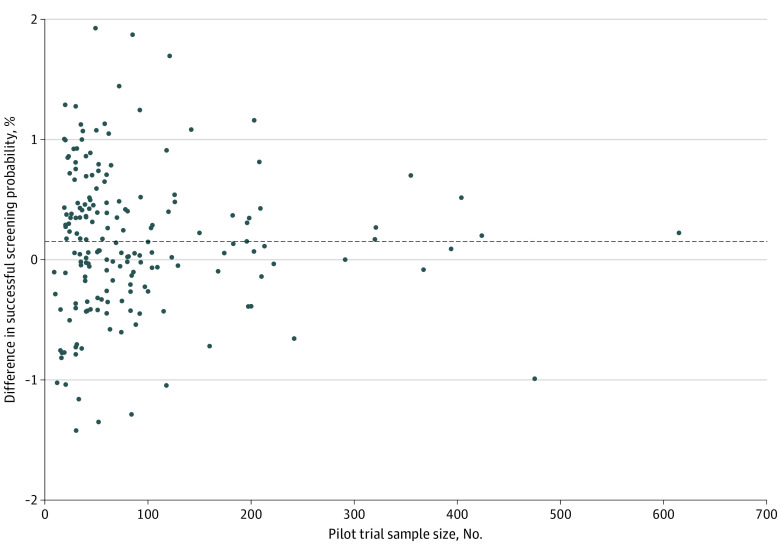
Scatterplot of Percentage Difference in Successful Screening Probability vs Pilot Trial Sample Size Dots represent the percentage difference, calculated by dividing the difference between the 2 studies (ie, pilot − full-scale) by their mean value. The dashed line represents the mean percentage difference.

Full-scale trials showed equivalent (35 full-scale trials) or improved (54 full-scale trials) successful screening in 89 of 183 pairs (43%). The likelihood of achieving equivalent or improved successful screening in full-scale trials was higher when the pilot trial utilized masking or blinding (RR, 1.41; 95% CI, 1.05-1.93) and feasibility progression criteria (RR, 1.94; 95% CI, 1.02-5.97) (Table 2). When the pilot trial was single center, the full-scale trial had higher likelihood of achieving an equivalent or improved successful screening if it was also conducted at a single center (RR, 1.50; 95% CI, 1.04-2.31) (Table 3). When participants or health care practitioners were masked in the full-scale trials, the likelihood of observing an equivalent or improved successful screening probability was higher if the pilot trial also masked the participants or health care practitioners compared with situations where they were unmasked (masked participants: RR, 1.82; 95% CI, 1.04-4.33; masked practitioners: RR, 1.81; 95% CI, 1.03-3.97) (Table 3).

**Table 2.** Association of Pilot Trial Characteristics With Concordance in Feasibility Estimates

Characteristic	Relative risk (95% CI)		
	Successful screening probability (n = 183)	Enrollment rate per week (n = 177)	Retention probability (n = 238)
General characteristics			
Disease^a^			
Addiction	1 [Reference]	1 [Reference]	1 [Reference]
Mental health	1.46 (0.75-3.75)	1.21 (0.85-1.85)	1.07 (0.86-1.38)
Obesity and physical activity	1.29 (0.59-3.36)	0.84 (0.41-1.43)	0.92 (0.67-1.24)
Oncology	1.58 (0.70-4.16)	0.92 (0.54-1.52)	1.08 (0.84-1.38)
Other	1.32 (0.78-3.27)	1.20 (0.91-1.82)	0.96 (0.81-1.24)
Intervention			
Behavioral	1 [Reference]	1 [Reference]	1 [Reference]
Pharmaceutical and others	1.18 (0.85-1.93)	1.03 (0.91-1.46)	1.30 (1.02-2.41)^b^
Publication year			
2004-2009	1 [Reference]	1 [Reference]	1 [Reference]
2010-2014	1.22 (0.81-1.97)	1.07 (0.88-1.33)	1.09 (0.94-1.28)
2015-2019	1.64 (1.11-2.62)^b^	0.95 (0.76-1.21)	1.07 (0.91-1.28)
Funding source			
Nonindustry	1 [Reference]	1 [Reference]	1 [Reference]
Industry	1.03 (0.39-1.74)	NA	NA
None or not reported	1.03 (0.53-1.60)	0.90 (0.54-1.20)	0.93 (0.69-1.15)
Cluster randomization			
No	1 [Reference]	1 [Reference]	1 [Reference]
Yes	0.93 (0.32-1.61)	1.11 (0.78-1.29)	0.89 (0.59-1.14)
Recruitment number			
Sites			
Single center	1 [Reference]	1 [Reference]	1 [Reference]
Multicenter	0.93 (0.73-1.37)	1.02 (0.90-1.46)	1.14 (0.97-1.89)
Groups			
2	1 [Reference]	1 [Reference]	1 [Reference]
>2	1.14 (0.85-2.30)	1.07 (0.94-3.64)	0.97 (0.95-1.47)
Sample size			
≤30	1 [Reference]	1 [Reference]	1 [Reference]
30-50	0.93 (0.53-1.62)	0.98 (0.74-1.29)	1.21 (1.03-1.44)^b^
50-100	1.38 (0.94-2.23)	1.07 (0.86-1.36)	1.10 (0.92-1.33)
>100	1.04 (0.63-1.76)	1.00 (0.79-1.28)	1.05 (0.86-1.30)
Sample size per group			
≤15	1 [Reference]	1 [Reference]	1 [Reference]
15-45	1.12 (0.78-1.72)	0.99 (0.81-1.24)	1.12 (0.97-1.31)
>45	1.03 (0.64-1.65)	1.04 (0.84-1.31)	1.05 (0.87-1.26)
Masking usage			
Masking used			
No	1 [Reference]	1 [Reference]	1 [Reference]
Yes	1.41 (1.05-1.93)^b^	1.03 (0.87-1.21)	1.04 (0.92-1.17)
Participants masked			
No	1 [Reference]	1 [Reference]	1 [Reference]
Yes	1.64 (1.20-2.19)^b^	0.96 (0.75-1.17)	1.05 (0.88-1.20)
Health care practitioner masked			
No	1 [Reference]	1 [Reference]	1 [Reference]
Yes	1.04 (0.39-1.76)	NA	0.87 (0.40-1.10)
Assessor masked			
No	1 [Reference]	1 [Reference]	1 [Reference]
Yes	1.10 (0.79-1.48)	1.10 (0.94-1.30)	1.00 (0.87-1.13)
Analyst masked			
No	1 [Reference]	1 [Reference]	1 [Reference]
Yes	1.91 (1.24-2.24)^b^	0.75 (0.35-1.11)	1.00 (0.67-1.16)
Outcome and aims			
Primary length of follow-up, mo	1.01 (0.97-1.03)	1.01 (1.00-1.04)	1.00 (0.99-1.01)
Intervention efficacy			
Not statistically significant	1 [Reference]	1 [Reference]	1 [Reference]
Statistically significant	1.16 (0.83-1.61)	1.12 (0.94-1.35)	1.00 (0.88-1.14)
Not evaluated	1.02 (0.59-1.56)	1.01 (0.78-1.27)	0.90 (0.73-1.08)
Pilot aim: efficacy			
No	1 [Reference]	1 [Reference]	1 [Reference]
Yes	0.96 (0.70-1.37)	0.91 (0.77-1.08)	1.07 (0.93-1.26)
Pilot aim: safety			
No	1 [Reference]	1 [Reference]	1 [Reference]
Yes	1.17 (0.84-1.75)	1.05 (0.88-1.27)	0.95 (0.85-1.08)
Pilot aim: feasibility			
No	1 [Reference]	1 [Reference]	1 [Reference]
Yes	1.03 (0.51-1.61)	1.03 (0.76-1.26)	1.07 (0.87-1.22)
Feasibility progression criteria used			
No	1 [Reference]	1 [Reference]	1 [Reference]
Yes	1.94 (1.02-5.97)^b^	0.96 (0.85-1.61)	0.94 (0.87-1.26)

Abbreviation: NA, not available.

^a^ The diseases listed represent the top 4 most frequently occurring within the data set. All other disease types are grouped under the category labeled as other. A complete list of diseases is available in eTable 2 in Supplement 1.

^b^ *P* < .05.

**Table 3.** Association of Modifications on Recruitment Number and Masking Usage With Concordance in Feasibility Estimates

Modifications compared with pilot	Relative risk (95% CI)		
	Successful screening probability (n = 183)	Enrollment rate per week (n = 177)	Retention probability (n = 238)
Recruitment No.			
Ratio of sample size per arm (pilot/full-scale)			
<50%	1 [Reference]	1 [Reference]	1 [Reference]
>50%	1.36 (0.78-1.95)	0.70 (0.31-1.08)	1.10 (0.87-1.22)
Effect size used for sample size calculation			
No	1 [Reference]	1 [Reference]	1 [Reference]
Yes	1.28 (0.93-1.72)	1.02 (0.83-1.21)	1.04 (0.91-1.18)
SD used for sample size calculation			
No	1 [Reference]	1 [Reference]	1 [Reference]
Yes	0.81 (0.61-1.30)	1.02 (0.88-1.72)	1.00 (0.90-1.61)
Difference in No. of sites	1.00 (0.98-1.00)	1.03 (1.01-1.08)^a^	1.00 (1.00-1.02)
No. of sites			
Pilot single center, full-scale multicenter	1 [Reference]	1 [Reference]	1 [Reference]
Both single center	1.50 (1.04-2.31)^a^	0.90 (0.74-1.10)	0.95 (0.82-1.11)
Both multicenter	1.20 (0.74-1.94)	0.97 (0.79-1.18)	1.06 (0.91-1.23)
No. of countries			
Same	1 [Reference]	1 [Reference]	1 [Reference]
More	1.66 (0.75-5.94)	1.17 (0.88-2.15)	NA
Masking usage			
No. of parties masked			
Same	1 [Reference]	1 [Reference]	1 [Reference]
More	0.97 (0.68-1.35)	1.00 (0.85-1.19)	1.05 (0.92-1.19)
Fewer	1.56 (0.997-2.19)	0.86 (0.52-1.18)	0.95 (0.69-1.18)
Participant masking status			
Pilot unmasked, full-scale masked	1 [Reference]	1 [Reference]	1 [Reference]
Both unmasked	1.15 (0.69-2.82)	1.36 (0.96-2.38)	0.90 (0.79-1.07)
Both masked	1.82 (1.04-4.33)^a^	1.39 (0.93-2.46)	0.94 (0.77-1.16)
Pilot masked, full-scale unmasked	2.06 (0.80-4.50)	0.85 (0.28-1.80)	0.98 (0.60-1.15)
Health care practitioner masking status			
Pilot unmasked, full-scale masked	1 [Reference]	1 [Reference]	1 [Reference]
Both unmasked	0.99 (0.60-2.20)	1.51 (1.02-2.83)^a^	0.95 (0.82-1.17)
Both masked	1.81 (1.03-3.97)^a^	1.55 (0.89-2.90)	1.08 (0.80-1.30)
Pilot masked, full-scale unmasked	NA	NA	NA
Assessor masking status			
Pilot unmasked, full-scale masked	1 [Reference]	1 [Reference]	1 [Reference]
Both unmasked	0.91 (0.62-1.35)	1.01 (0.82-1.27)	0.92 (0.80-1.06)
Both masked	0.98 (0.64-1.49)	1.07 (0.85-1.35)	1.00 (0.86-1.16)
Pilot masked, full-scale unmasked	1.23 (0.63-1.99)	NA	0.75 (0.46-1.03)
Analyst masking status			
Pilot unmasked, full-scale masked	1 [Reference]	1 [Reference]	1 [Reference]
Both unmasked	0.75 (0.52-1.24)	0.85 (0.75-1.06)	1.10 (0.89-1.47)
Both masked	1.11 (0.42-1.78)	0.74 (0.28-1.05)	0.80 (0.29-1.26)
Pilot masked, full-scale unmasked	NA	0.56 (0.19-0.96)^a^	NA

Abbreviation: NA, not available.

^a^ *P* < .05.

### Enrollment Rate

The median (IQR) overall enrollment rate among 177 trial pairs was 1.7 (0.6 to 5.4) participants per week for pilot trials and 2.9 (1.3 to 8.5) participants per week for full-scale trials. The mean (SD) percentage difference between the 2 was −52% (85; median (IQR): −59% [−121% to – 4%]). For the site-average enrollment rate, pilot trials had a median (IQR) rate of 1.2 (0.5 to 3.3) participants per week per site, while full-scale trials had a median (IQR) rate of 1.0 (0.4 to 3.3) participants per week per site. The mean (SD) percentage difference was 7% (92%; median [IQR]: 7% [−56% to 83%]).

Of 177 pairs, 136 (77%) had equivalent (9 full-scale trials) or improved (127 full-scale trials) overall enrollment rates in the full-scale trial compared with the pilot trial. Having 1 more study site in the full-scale trial was associated with 1.03 (95% CI, 1.01-1.08) times higher likelihood of equivalent or improved enrollment rates (Table 3). When health care practitioners were unmasked in the pilot trial, the full-scale trial had higher likelihood of achieving an equivalent or improved enrollment rate if trialists did not change this design feature (RR, 1.51; 95% CI, 1.02-2.83) (Table 3). However, modifying the intervention was associated with lower likelihood of equivalent or improved enrollment rates (RR, 0.84; 95% CI, 0.70-0.99), as was extending the length of follow-up in the full-scale trial (RR, 0.81; 95% CI, 0.68-0.96) (Table 4).

**Table 4.** Association of Modifications on PICO Components With Concordance in Feasibility Estimates

Modifications compared with pilot	Relative risk (95% CI)		
	Successful screening probability (n = 183)	Enrollment rate per week (n = 177)	Retention probability (n = 238)
Population (P)			
Eligibility modified			
No	1 [Reference]	1 [Reference]	1 [Reference]
Yes	0.83 (0.61-1.13)	0.98 (0.83-1.17)	0.99 (0.88-1.14)
Eligibility modification type			
Broader	1 [Reference]	1 [Reference]	1 [Reference]
Narrower	0.89 (0.51-1.39)	0.91 (0.70-1.14)	1.04 (0.88-1.23)
Same	0.96 (0.68-1.39)	0.92 (0.77-1.10)	1.00 (0.87-1.17)
Intervention (I)			
No. of groups			
Same	1 [Reference]	1 [Reference]	1 [Reference]
More	0.78 (0.33-1.33)	1.08 (0.79-1.29)	1.00 (0.78-1.18)
Fewer	1.01 (0.57-1.49)	0.96 (0.63-1.23)	0.98 (0.76-1.17)
Intervention modification type			
Same	1 [Reference]	1 [Reference]	1 [Reference]
Modified	0.93 (0.67-1.26)	0.84 (0.70-0.99)^a^	0.97 (0.84-1.09)
Other difference	0.43 (0.16-1.07)	0.85 (0.46-1.12)	0.99 (0.65-1.16)
Intervention modified			
No	1 [Reference]	1 [Reference]	1 [Reference]
Yes	0.88 (0.63-1.18)	0.84 (0.70-0.99)^a^	0.97 (0.85-1.09)
Intervention content			
Same	1 [Reference]	1 [Reference]	1 [Reference]
Added content	0.74 (0.45-1.07)	1.00 (0.81-1.20)	0.82 (0.66-0.98)^a^
Reduced content	0.37 (0.21-1.22)	0.51 (0.19-1.01)	1.03 (0.64-1.12)
Intervention duration			
Same	1 [Reference]	1 [Reference]	1 [Reference]
Longer duration	1.12 (0.75-1.54)	0.85 (0.63-1.08)	1.00 (0.84-1.15)
Shorter duration	1.24 (0.44-1.86)	0.94 (0.40-1.11)	NA
Intervention frequency			
Same	1 [Reference]	1 [Reference]	1 [Reference]
More frequent	0.80 (0.29-1.60)	0.64 (0.30-1.00)	1.02 (0.51-1.13)
Less frequent	0.99 (0.46-1.57)	0.64 (0.30-1.00)	NA
Comparison (C)			
Control modified			
No	1 [Reference]	1 [Reference]	1 [Reference]
Yes	1.15 (0.83-1.56)	1.01 (0.84-1.19)	0.99 (0.86-1.12)
Control modification type			
Modified	1 [Reference]	1 [Reference]	1 [Reference]
Same	0.89 (0.60-1.50)	0.97 (0.80-1.23)	0.93 (0.82-1.09)
Active in main, placebo in pilot	0.96 (0.44-1.83)	1.03 (0.66-1.35)	0.74 (0.48-0.99)^a^
Placebo in main, active in pilot	1.28 (0.50-2.25)	0.63 (0.23-1.08)	0.71 (0.29-1.03)
Other difference	1.04 (0.46-1.98)	0.97 (0.63-1.33)	1.07 (0.86-1.22)
Control content			
Same	1 [Reference]	1 [Reference]	1 [Reference]
Added content	1.33 (0.69-1.99)	1.11 (0.79-1.29)	1.06 (0.82-1.20)
Reduced content	0.72 (0.36-1.68)	1.05 (0.44-1.20)	1.01 (0.49-1.11)
Control duration			
Same	1 [Reference]	1 [Reference]	1 [Reference]
Longer duration	0.85 (0.32-1.70)	0.64 (0.22-1.08)	NA
Shorter duration	1.41 (0.51-1.88)	NA	NA
Control frequency			
Same	1 [Reference]	1 [Reference]	1 [Reference]
More frequent	NA	0.65 (0.31-1.01)	0.60 (0.29-0.93)^a^
Less frequent	NA	NA	NA
Outcome (O)			
Length of follow-up (longest)			
Same	1 [Reference]	1 [Reference]	1 [Reference]
Longer	0.95 (0.68-1.38)	0.81 (0.68-0.96)^a^	0.93 (0.81-1.06)
Shorter	0.78 (0.33-1.38)	0.94 (0.71-1.14)	0.99 (0.78-1.16)
Length of follow-up (primary)			
Same	1 [Reference]	1 [Reference]	1 [Reference]
Different	1.32 (0.98-1.78)	0.84 (0.69-0.99)^a^	0.85 (0.73-0.97)^a^
No. of follow-up visits			
Same	1 [Reference]	1 [Reference]	1 [Reference]
More	0.93 (0.66-1.27)	1.01 (0.84-1.22)	0.86 (0.75-0.98)^a^
Fewer	0.87 (0.40-1.43)	1.20 (0.94-1.41)	0.94 (0.74-1.10)

Abbreviations: NA, not available; PICO, patient/population/problem, intervention, comparison, outcome.

^a^ *P* < .05.

### Retention Probability

The retention probability among 238 trial pairs was found to be similar for both pilot and full-scale trials, with a mean (SD) of 83.5% (15) and 84.2% (13%), respectively (mean [SD] percentage difference, −1% [19]; median [IQR], 0%, [−9% to 6%]) (eFigure 2 in Supplement 1).

Approximately 82% of full-scale trials (194 of 238) achieved an equivalent (138 full-scale trials) or improved (56 full-scale trials) retention probability. If the pilot trial had a sample size between 30 and 50, the retention probability had higher likelihood of being equivalent or improved compared with pilot trials with a sample size of less than 30 (RR, 1.21; 95% CI, 1.03-1.44) (Table 2). The likelihood of having an equivalent or improved retention probability was lower if the full-scale trial added extra content to the intervention (RR, 0.82; 95% CI, 0.66-0.98), changed the comparison group from placebo or no treatment to active control as opposed to simple modification (RR, 0.74; 95% CI, 0.48-0.99), administrated the control intervention more frequently (RR, 0.60; 95% CI, 0.29-0.93), had a different length of follow-up (RR, 0.85; 95% CI, 0.73-0.97), or conducted more follow-up visits (RR, 0.86; 95% CI, 0.75-0.98) (Table 4).

## Discussion

This study first compared feasibility estimates between pilot and full-scale trials. On average, screening success was slightly lower (7%) in full-scale trials, with only 43% of trials showing improved screening. However, 77% of full-scale trials had better enrollment rates (mean increase of 52%). Estimated retention probability had good agreement between pilot and full-scale trials, with a 1% difference and more than half of the values within the 10% equivalence margin. This aligns with a previous study comparing 16 pairs of pilot and full-scale trials.^10^

The observed decrease in screening proportion and increase in enrollment rates in full-scale trials could be attributed to the greater number of study sites compared with pilot trials. While multisite trials can expedite enrollment through simultaneous recruitment at different sites, they also face a more diverse participant pool. This diversity may lower the screening proportion, as not all seemingly eligible participants ultimately qualify. Our associational analysis indeed showed that trials with more sites than their pilot often achieved higher enrollment, but multicenter full-scale trials following single-center pilots had lower likelihood of similar or improved screening success. Therefore, researchers conducting full-scale trials at multiple sites may anticipate faster recruitment but should also prepare for a larger screening pool to reach the target sample size.

Masking has been widely recognized as a factor that can hinder study recruitment.^17,26,27,28^ We found that masking was one of the few design features in pilot trials that was associated with an equivalent or improved probability of successful screening. Our results also suggest that if masking is envisioned in the full-scale trial, it is desirable to use it in the pilot trial. We recommend that the pilot and full-scale trials be consistent in terms of masking to maximize recruitment feasibility.

Our analyses also found that protocol modifications may decrease the feasibility of full-scale trials if they impose a greater burden on participants. Such modifications include additional intervention content, changing the comparator from placebo or no treatment to active treatment, administrating the control treatment more frequently, prolonged follow-up periods, and increasing the number of follow-up visits. Previous qualitative and quantitative evidence has suggested that potential trial participants may perceive high time commitments and demanding follow-up schedules as too burdensome,^29,30,31,32^ leading to increased screening failure and dropouts.^33^ Quantifying participant burden and incorporating it into the study protocol to evaluate feasibility has been suggested.^34,35,36^

It has been recommended that pilot trials incorporate prespecified progression criteria to aid in the decision-making process for proceeding with a full-scale trial.^7^ Typically, these criteria set a threshold above which the full-scale trial is deemed feasible. The decision to proceed can be made in a binary fashion by comparing the feasibility parameter’s point estimate to the threshold or by testing whether the confidence interval around the estimate includes the threshold. While progression criteria have been used in research practice,^37^ few studies have investigated whether their use improves the performance of pilot trials in informing the feasibility of full-scale trials. Our analysis suggests that using feasibility progression criteria in the pilot trial may result in an equivalent or improved probability of successful screening in the full-scale trial. However, we did not observe a similar association for recruitment rate or retention probability. Further examination of the data revealed that this difference may be attributed to subsequent modifications made to the trial design. These modifications were associated with worse retention probability and recruitment rate, while maintaining or enhancing screening probability. Our findings imply that the utility of progression criteria might be undermined by modifications made after the pilot phase.

In the current study, we adopted a broad definition of pilot trials, not excluding studies solely due to the implementation of effect size estimation or hypothesis testing, despite concerns that have been raised about these practices.^2,3,4,5,6^ This approach is partially based on the understanding that treatment efficacy could affect trial retention and participant recruitment. We also presumed that studies, even if not explicitly assessing feasibility, inherently do so during execution. Nonetheless, pilot trials primarily focusing on efficacy estimation were excluded at the analysis stage if they did not report 3 feasibility parameters of interest.

### Limitations

This study has limitations. First, we did not differentiate between true pilot trials and those potentially mislabeled. However, we posit that post hoc mislabeling of studies as pilot trials to excuse small sample sizes, low methodological quality, or incomplete studies is less likely in our data set, considering all studies informed a full-scale trial. Second, by excluding pilot trials not followed by full-scale trials, we may have observed an attenuated association. The absence of a full-scale trial may indicate its infeasibility even with significant modifications. The association between trial modifications and feasibility would be stronger in such cases because the modifications altered the full-scale trial from being infeasible to feasible. Third, we used a complete case analysis, excluding pairs with missing feasibility estimates. This nonreporting indicates a lack of adherence to the Consolidated Standards of Reporting Trials (CONSORT) guidelines^38^ and possibly inferior methodological quality, as reporting quality often proxies for methodological quality.^39^ Fourth, there are other important factors that can affect trial recruitment and retention, such as the use of incentives and the follow-up format,^17,18^ which we were not able to examine in our study. Fifth, multiple trial aspects may be modified simultaneously, and these modifications may influence the feasibility of the full-scale trial in different ways and magnitudes. Additionally, the current study examined various characteristics. However, per nature of its design, the width of the confidence intervals was not adjusted for multiple comparisons, and the results should be viewed as exploratory.

## Conclusions

Using pilot trial estimates to inform the full-scale trial’s feasibility can be challenging due to biases introduced by modifications and random errors magnified by the small sample size. While the agreement between pilot and full trials may improve with larger sample sizes, systematic errors may still persist. Trialists and funders should consider potential impacts of protocol modifications on feasibility when planning or assessing a full-scale trial. On average, full-scale trials had slightly lower screening success, better enrollment rates, and comparable retention probabilities than the pilot trial. Consistency in masking is desirable, and the pilot trial’s use of feasibility progression criteria might improve full-scale trial feasibility. Modifications that increase participant burden may make full-scale trials less feasible.

## Notes

Supplement 1. **eTable 1. **Search Strategy **eFigure 1.** Flowchart of Study Selection Process **eAppendix.** Missing Data Description **eTable 2.** Complete List of Diseases **eTable 3.** Comparison of Pilot Trial Characteristics Between Pairs With and Without Missing Data on Feasibility Parameters **eTable 4.** Comparison of Full-Scale Trial Characteristics Between Pairs With and Without Missing Data on Feasibility Parameters **eFigure 2.** Scatterplot of Percentage Difference in Retention Probability vs Pilot Trial Sample Size

Supplement 2.
Data Sharing Statement
