# Supplementary material for: Pilot Trial Characteristics, Postpilot Design Modifications, and Feasibility of Full-Scale Trials
Source: JAMA Netw Open. 2023 Sep 14;6(9):e2333642. doi: 10.1001/jamanetworkopen.2023.33642 (PMC10502523; doi:10.1001/jamanetworkopen.2023.33642)
Supplement: Supplement 1. — eTable 1. Search Strategy eFigure 1. Flowchart of Study Selection Process eAppendix. Missing Data Description eTable 2. Complete List of Diseases eTable 3. Comparison of Pilot Trial Characteristics Between Pairs With and Without Missing Data on Feasibility Parameters eTable 4. Comparison of Full-Scale Trial Characteristics Between Pairs With and Without Missing Data on Feasibility Parameters eFigure 2. Scatterplot of Percentage Difference in Retention Probability vs Pilot Trial Sample Size [file jamanetwopen-e2333642-s001.pdf]

Supplemental Online Content

Ying X, Ehrhardt S. Pilot trial characteristics, postpilot design modifications, and feasibility of full-scale trials. *JAMA Netw Open.* 2023;6(9):e2333642. doi:10.1001/jamanetworkopen.2023.33642

eTable 1. Search Strategy

eFigure 1. Flowchart of Study Selection Process

eAppendix. Missing Data Description

eTable 2. Complete List of Diseases

eTable 3. Comparison of Pilot Trial Characteristics Between Pairs With and Without Missing Data on Feasibility Parameters

eTable 4. Comparison of Full-Scale Trial Characteristics Between Pairs With and Without Missing Data on Feasibility Parameters

eFigure 2. Scatterplot of Percentage Difference in Retention Probability vs Pilot Trial Sample Size

This supplemental material has been provided by the authors to give readers additional information about their work.

© 2023 Ying X et al. *JAMA Network Open.*

eTable 1. Search Strategy

#1	"Pilot Projects"[Mesh] OR "Feasibility Studies"[Mesh]
#2	(Feasib*[Title/Abstract] OR pilot[Title/Abstract]) AND (study[Title/Abstract] OR trial[Title/Abstract])
#3	#1 OR #2
#4	retention[Title/Abstract] OR attrition[Title/Abstract] OR recruitment[Title/Abstract] OR randomization[Title/Abstract] OR participation[Title/Abstract] OR adherence[Title/Abstract] OR compliance[Title/Abstract] OR acceptability[Title/Abstract] OR completion[Title/Abstract] OR attendance[Title/Abstract]
#5	randomized controlled trial[pt] OR controlled clinical trial[pt] OR randomized[tiab] OR placebo[tiab] OR drug therapy[sh] OR randomly[tiab] OR trial[tiab] OR groups[tiab] NOT (animals [mh] NOT humans [mh])
#6	random*[Title/Abstract]
#7	#5 AND #6
#8	#3 AND # 4 AND #7

© 2023 Ying X et al. *JAMA Network Open.*

eFigure 1. Flowchart of Study Selection Process

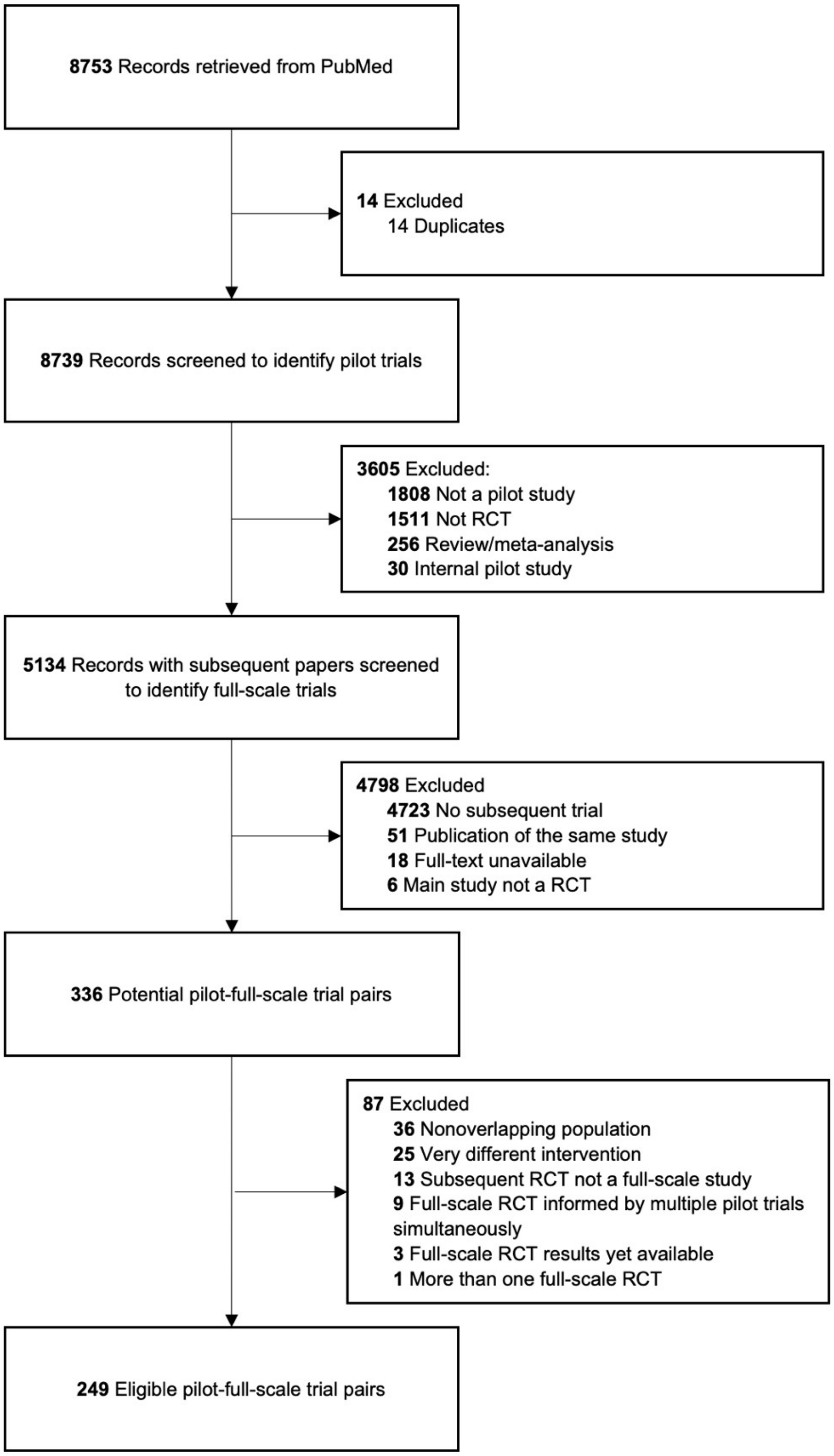

© 2023 Ying X et al. *JAMA Network Open.*

eAppendix. Missing Data Description

Information on successful screening probability, enrollment rate, and retention probability was available in 183, 177, and 238 pairs of pilot and full-scale trials, respectively. The primary source of this missing data was the pilot trials. Specifically, successful screening probability was not reported in 57 pilot trials and 30 full-scale trials. Enrollment rate was omitted in 69 pilot trials and 20 full-scale trials, while retention probability was not reported in 11 pilot and 11 full-scale trials. Comparison of pilot and full-scale trial characteristics between pairs with and without missing data is available in eTable 3 and eTable 4.

Pilot trials from pairs with unavailable data on successful screening probability were less likely to have more than two arms (8% vs 19%, P=.035), while tending to have larger average sample sizes (212±554 vs 88±93, P=.004) (eTable 3). No significant between-group differences were found in relation to the full-scale trials' characteristics (eTable 4).

Regarding the enrollment rate, pilot trials in pairs lacking data on this metric more commonly examined interventions for obesity or physical activity (18% vs 8%, P=.047), were less frequently published post-2015 (14% vs 35%, P=0.003), and were less likely to use masking (33% vs 49%, P=.028) (eTable 3). The full-scale trials from pairs lacking data on the enrollment rate were also less frequently published after 2015 (18% vs 32%, P=.031), had a higher likelihood of being cluster-randomized trials (25% vs 12%, P=.010), were less frequently multicenter (38% vs 60%, P<.001), and had smaller median sample sizes (203 vs 290, P=.024) (eTable 4).

Pilot trials in pairs missing data on retention probability had larger median sample sizes (479 vs 104, P<.001) and longer median follow-up lengths (182 vs 91 days, P=.038) (eTable 3). Similarly, full-scale trials in pairs missing data on retention probability also had larger median sample sizes (600 vs 264, P=.016) and longer median follow-up lengths (365 vs 182 days, P=.019) (eTable 4).

eTable 2. Complete List of Diseases

Disease	Freq.	Percent
mental health	34	13.65
addiction	24	9.64
oncology	21	8.43
physical activity	14	5.62
obesity	13	5.22
pain	12	4.82
HIV	11	4.42
stroke	11	4.42
orthopedics	10	4.02
aging	7	2.81
diabetes	7	2.81
heart disease	7	2.81
multiple sclerosis	6	2.41
diet	5	2.01
healthcare	5	2.01
obstetric	5	2.01
sleep	5	2.01
developmental	4	1.61
transplantation	4	1.61
acute respiratory infection	3	1.2
critical care	3	1.2
neuropathy	3	1.2
parenting	3	1.2
renal	3	1.2
chronic obstructive pulmonary disease	2	0.8
dementia	2	0.8
hypertension	2	0.8
metabolic	2	0.8
Duchenne Muscular Dystrophy	1	0.4
acute lung injury	1	0.4
asthma	1	0.4
auditory hallucination	1	0.4
bacteremia	1	0.4
Barrett esophagus	1	0.4
blood donation	1	0.4
chronic fatigue syndrome	1	0.4
cystic fibrosis	1	0.4
diarrhea	1	0.4

© 2023 Ying X et al. *JAMA Network Open.*

domestic violence	1	0.4
gonorrhoeae	1	0.4
irritable bowel syndrome	1	0.4
otitis media prophylaxis	1	0.4
Parkinson	1	0.4
seizure	1	0.4
spinal cord injury	1	0.4
tuberculosis	1	0.4
urinary tract infection	1	0.4
vaccination	1	0.4
vitiligo	1	0.4
Total	249	100

© 2023 Ying X et al. *JAMA Network Open.*

eTable 3. Comparison of Pilot Trial Characteristics Between Pairs With and Without Missing Data on Feasibility Parameters[a]

	No. (%)								
	Successful screening probability			Enrollment rate per week			Retention probability		
	Non-missing (n=183)	Missing (n=66)	P-value	Non-missing (n=177)	Missing (n=72)	P-value	Non-missing (n=238)	Missing (n=11)	P-value
Disease [b]			0.45			**0.047**			0.33
Addiction	19 (10)	5 (8)		**19 (11)**	**5 (7)**		23 (10)	1 (9)	
Mental health	26 (14)	8 (12)		**23 (13)**	**11 (15)**		34 (14)	0 (0)	
Obesity & physical activity	21 (11)	6 (9)		**14 (8)**	**13 (18)**		25 (11)	2 (18)	
Oncology	12 (7)	9 (14)		19 (11)	2 (3)		19 (8)	2 (18)	
Other	105 (57)	38 (58)		102 (58)	41 (57)		137 (58)	6 (55)	
Intervention			0.15			0.11			0.51
Behavioral	131 (72)	41 (62)		117 (66)	55 (76)		163 (68)	9 (82)	
Pharmaceutical & other	52 (28)	25 (38)		60 (34)	17 (24)		75 (32)	2 (18)	
Publication year			0.32			**0.003**			0.93
2004-2009	50 (27)	24 (36)		**46 (26)**	**28 (39)**		70 (29)	4 (36)	
2010-2014	80 (44)	23 (35)		**69 (39)**	**34 (47)**		99 (42)	4 (36)	
2015-2019	53 (29)	19 (29)		**62 (35)**	**10 (14)**		69 (29)	3 (27)	
Funding source			0.75			0.22			0.45
Non-industry	161 (88)	59 (89)		160 (90)	60 (83)		211 (89)	9 (82)	
Industry	4 (2)	2 (3)		4 (2)	2 (3)		6 (3)	0 (0)	
None or not reported	18 (10)	5 (8)		13 (7)	10 (14)		21 (9)	2 (18)	
Cluster randomization			0.40			0.78			0.17
No	172 (94)	60 (91)		164 (93)	68 (94)		223 (94)	9 (82)	
Yes	11 (6)	6 (9)		13 (7)	4 (6)		15 (6)	2 (18)	
No. of sites			0.92			0.093			0.73
Single center	137 (75)	49 (74)		127 (72)	59 (82)		177 (74)	9 (82)	
Multicenter	46 (25)	17 (26)		50 (28)	13 (18)		61 (26)	2 (18)	
No. of arms			**0.035**			0.30			0.68
2	**149 (81)**	**61 (92)**		152 (86)	58 (81)		201 (84)	9 (82)	
>2	**34 (19)**	**5 (8)**		25 (14)	14 (19)		37 (16)	2 (18)	
Sample size									
Mean (SD)	**88 (93)**	**212 (554)**	**0.004**	143 (351)	67 (60)	0.072	**104 (213)**	**479 (1002)**	**<0.001**
Median (IQR)	56 (34, 100)	49 (28, 100)	0.35	60 (32, 115)	48 (30, 82)	0.11	52 (31, 100)	66 (33, 355)	0.32
Masking used			0.96			**0.028**			0.36
No	102 (56)	37 (56)		**91 (51)**	**48 (67)**		131 (55)	8 (73)	
Yes	81 (44)	29 (44)		**86 (49)**	**24 (33)**		107 (45)	3 (27)	

© 2023 Ying X et al. *JAMA Network Open.*

Primary length of follow-up (days)									
Mean (SD)	158 (236)	187 (240)	0.39	178 (263)	136 (152)	0.21	163 (240)	220 (144)	0.44
Median (IQR)	91 (45, 182)	91 (30, 274)	0.87	91 (61, 182)	91 (14, 182)	0.22	**91 (42, 182)**	**182 (91, 365)**	**0.038**
Intervention efficacy			0.13			0.78			0.31
Not statistically significant	81 (44)	28 (42)		79 (45)	30 (42)		106 (45)	3 (27)	
Statistically significant	72 (39)	20 (30)		63 (36)	29 (40)		88 (37)	4 (36)	
Not evaluated	30 (16)	18 (27)		35 (20)	13 (18)		44 (18)	4 (36)	

Abbreviations: SD, standard deviation; IQR, interquartile range.

[a] P values are derived from Student's t tests for means, Wilcoxon rank-sum tests for medians, Pearson's Chi-squared tests for frequencies if all cell counts exceed 5 or Fisher's Exact tests if at least one cell count is less than 5. Bold text indicates P<.05.

[b] The diseases listed represent the top four most frequently occurring within the dataset. All other disease types are grouped under the category labeled as "other." A complete list of diseases is available in eTable 2 in the Supplement.

© 2023 Ying X et al. *JAMA Network Open.*

eTable 4. Comparison of Full-Scale Trial Characteristics Between Pairs With and Without Missing Data on Feasibility Parameters[a]

	No. (%)								
	Successful screening probability			Enrollment rate per week			Retention probability		
	Non-missing (n=183)	Missing (n=66)	P-value	Non-missing (n=183)	Missing (n=66)	P-value	Non-missing (n=183)	Missing (n=66)	P-value
Publication year			0.61			**0.031**			0.51
2004-2009	4 (2)	2 (3)		**2 (1)**	**4 (6)**		6 (3)	0 (0)	
2010-2014	34 (19)	17 (26)		**33 (19)**	**18 (25)**		47 (20)	4 (36)	
2015-2019	93 (51)	30 (45)		**86 (49)**	**37 (51)**		119 (50)	4 (36)	
2020-2022	52 (28)	17 (26)		**56 (32)**	**13 (18)**		66 (28)	3 (27)	
Funding source			0.45			0.55			0.34
Non-industry	171 (93)	59 (89)		165 (93)	65 (90)		220 (92)	10 (91)	
Industry	7 (4)	5 (8)		7 (4)	5 (7)		12 (5)	0 (0)	
None or not reported	5 (3)	2 (3)		5 (3)	2 (3)		6 (3)	1 (9)	
Cluster randomization			0.29			**0.010**			0.075
No	157 (86)	53 (80)		**156 (88)**	**54 (75)**		203 (85)	7 (64)	
Yes	26 (14)	13 (20)		**21 (12)**	**18 (25)**		35 (15)	4 (36)	
No. of sites			0.47			**<0.001**			0.96
Single center	82 (45)	33 (50)		**70 (40)**	**45 (62)**		110 (46)	5 (45)	
Multicenter	101 (55)	33 (50)		**107 (60)**	**27 (38)**		128 (54)	6 (55)	
No. of arms			0.60			0.51			0.075
2	153 (84)	57 (86)		151 (85)	59 (82)		203 (85)	7 (64)	
>2	30 (16)	9 (14)		26 (15)	13 (18)		35 (15)	4 (36)	
Sample size									
Mean (SD)	935 (2562)	1800 (6750)	0.14	1470 (4833)	414 (574)	0.066	**842 (2005)**	**8145 (16364)**	**<0.001**
Median (IQR)	269 (140, 560)	256 (150, 861)	0.43	**290 (150, 697)**	**203 (132, 373)**	**0.024**	**264 (140, 599)**	**600 (250, 11880)**	**0.016**
Masking used			0.22			0.082			0.062
No	62 (34)	28 (42)		58 (33)	32 (44)		83 (35)	7 (64)	
Yes	121 (66)	38 (58)		119 (67)	40 (56)		155 (65)	4 (36)	
Primary length of follow-up (days)									
Mean (SD)	330 (465)	295 (304)	0.56	342 (482)	270 (243)	0.23	**307 (377)**	**616 (1032)**	**0.019**

© 2023 Ying X et al. *JAMA Network Open.*

Median (IQR)	210 (91, 365)	182 (90, 365)	0.62	182 (91, 365)	182 (91, 365)	0.57	182 (91, 365)	365 (84, 548)	0.26
Intervention efficacy			0.55			0.082			**0.043**
Not statistically significant	84 (46)	35 (53)		90 (51)	29 (40)		**115 (48)**	**4 (36)**	
Statistically significant	98 (54)	31 (47)		87 (49)	42 (58)		**123 (52)**	**6 (55)**	
Not evaluated	1 (1)	0 (0)		0 (0)	1 (1)		**0 (0)**	**1 (9)**	

Abbreviations: SD, standard deviation; IQR, interquartile range.

[a] P values are derived from Student's t tests for means, Wilcoxon rank-sum tests for medians, Pearson's Chi-squared tests for frequencies if all cell counts exceed 5 or Fisher's Exact tests if at least one cell count is less than 5. Bold text indicates P<.05.

© 2023 Ying X et al. *JAMA Network Open.*

eFigure 2. Scatterplot of Percentage Difference in Retention Probability vs Pilot Trial Sample Size

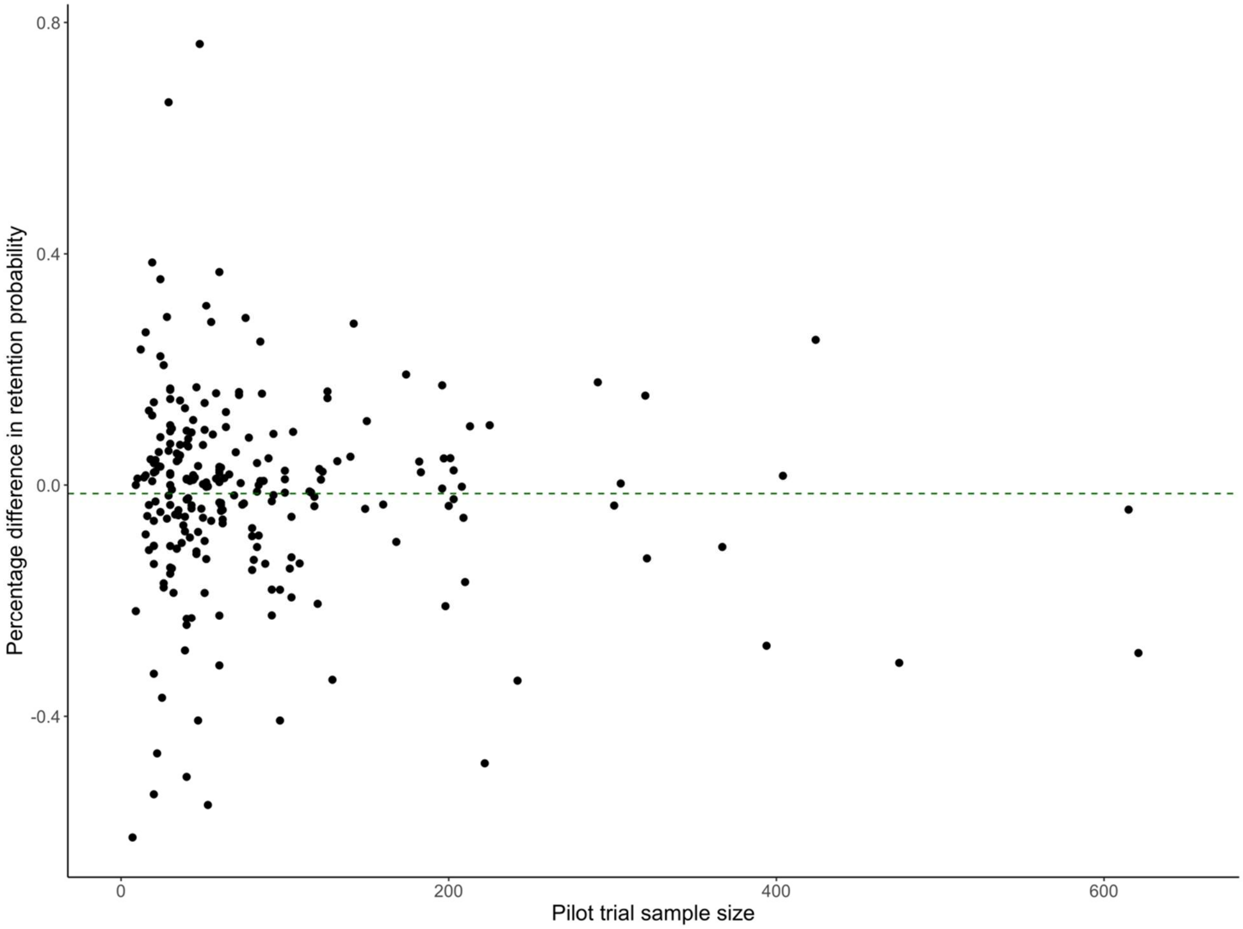

Dots represent the percentage difference, calculated by dividing the difference between the two studies (i.e., pilot - full-scale) by their mean value. The dashed line represents the average percentage difference.

© 2023 Ying X et al. *JAMA Network Open.*
