# Supplementary material for: Pilot Trial Characteristics, Postpilot Design Modifications, and Feasibility of Full-Scale Trials
Source: JAMA Netw Open. 2023 Sep 14;6(9):e2333642. doi: 10.1001/jamanetworkopen.2023.33642 (PMC10502523; doi:10.1001/jamanetworkopen.2023.33642)
Supplement: Supplement 2. — Data Sharing Statement [file jamanetwopen-e2333642-s002.pdf]

Data Sharing Statement

Ying. Pilot Trial Characteristics, Postpilot Design Modifications, and Feasibility of Full-Scale Trials. *JAMA Netw Open*. Published September 14, 2023.
doi:10.1001/jamanetworkopen.2023.33642

Data
Data available: Yes
Data types: Data (not involving human participants)
How to access data: For accessing the data, please contact the first author at xying5@jh.edu.
When available: With publication

Supporting Documents
Document types: None

Additional Information
Who can access the data: Data is available for sharing upon reasonable request by contacting the authors.
Types of analyses: Data is available for sharing upon reasonable request by contacting the authors.
Mechanisms of data availability: Data is available for sharing upon reasonable request by contacting the authors.
